# Prevalence of fibromyalgia and irritable bowel syndrome and its association with studying medicine, a cross‐sectional study in Al‐Baath University, Syria

**DOI:** 10.1002/brb3.3445

**Published:** 2024-03-11

**Authors:** Mohanad Daher, Sara Abbas, Zainab Asaad, Karam Khalil, Ghania Jadid

**Affiliations:** ^1^ Faculty of Medicine Damascus University Damascus Syria; ^2^ Scientific Representative at Faculty of Medicine Al‐Baath University Homs Syria

**Keywords:** fibromyalgia, psychiatric, stress, syndrome, irritable bowel

## Abstract

**Background:**

The aim of this study is to assess the prevalence rate of fibromyalgia (FM) and irritable bowel syndrome (IBS) among Al‐Baath University students and find out whether studying medicine has an association with a higher prevalence rate.

**Methods:**

The participants of this observational cross‐sectional study were students aged 18–30 years from Al‐Baath University. A structured self‐estimated electronic questionnaire developed by Google Forms was distributed using social media platforms from 15 February to 15 March, 2023. We used The American College of Rheumatology (ACR) 2016 and Fibromyalgia Rapid Screening Tool criteria to assess the prevalence rate of FM. We used The ROME IV criteria to asses IBS prevalence rate.

**Results:**

The final sample size was 800 individuals. The prevalence of IBS in the study population was 26.8%. Overall, IBS‐Constipation was the most common subtype, and the prevalence rate was higher among medical students (14%) compared to other colleges (12.8%) (*p* = .002). The difference in IBS prevalence between males and females was (9.3% vs. 17.5%, *p* = .283), but this did not reach the statistical significance. The prevalence of FM according to The ACR in the study population was 3.6%. Females had higher prevalence rate than males (3.1% vs. 0.5%, *p* = .007). The prevalence of FM was also higher in other colleges compared to medicine (2.3% vs. 1.4%, *p* = .547), but this did not reach statistical significance.

**Conclusion:**

We found an increased prevalence of IBS among medical students. The prevalence of FM did not show any relation to studying medicine. We recommend additional prospective studies to assess whether studying medicine could be a risk factor for these disorders or not.

## Graphical abstract

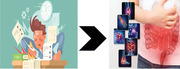

## 1 INTRODUCTION

The American College of Rheumatology (ACR) identifies fibromyalgia (FM) as suffering severe widespread pain and tenderness in at least 11 or more of 18 specific tender spots for at least 3 months (Wolfe et al., 1990). Several clinical findings, including chronic headaches, paresthesia, sleeping problems, cognitive and mood impairments, psychiatric manifestations (depression, anxiety), and visceral pain, may also be present (Ioachim et al., 2022; Sarzi‐Puttini et al., 2020). According to the diagnostic criteria that were applied, the prevalence of FM ranges from 0.2% to 6.6% globally (Cabo‐Meseguer et al., 2017; Jones et al., 2015). Females are three times more likely to have FM than males (Cabo‐Meseguer et al., 2017). Although there are many factors known to affect the prevalence of FM, the exact pathophysiology of this disease is still relatively unknown. FM prevalence has been linked to childhood difficulties, female sex, smoking, high body mass index (BMI), alcohol abstinence, and preexisting medical disorders in adulthood (Creed, 2020). One factor that has been demonstrated to have a significant effect is stress, and several studies have looked at how different kinds of stress can trigger the development of FM (Lumley et al., 2011). Irritable bowel syndrome (IBS) is the most common functional gastrointestinal disorder (Lacy & Patel, 2017). Rome foundation identifies IBS as having a recurrent abdominal pain related to defecation in the last 3 months associated with a change in frequency and form of stool (Rome Foundation). IBS was found to be a combination of irritable bowel and irritable brain; the gut is affected by changes in neuroendocrine–immune pathways brought on by stress, which significantly affect the permeability, secretion, motility, and sensitivity of the intestines, and that is what induces an amplification or flare‐up of IBS symptoms (Qin et al., 2014). Functional syndromes like IBS, FM, and other psychiatric disorders interact in complex ways, and usually coexist, that suggest a common pathogenetic mechanism for FM and IBS (Veale et al., 1991). According to studies, stress levels among medical students are elevated particularly during their undergraduate course (Rosal et al., 1997; Styles, 1993). Excessive workload, difficulties with studying and time management, conflicts in work–life balance, and relationships, medical school peer relations, health concerns, and financial stressors were noted as significant factors in the prevalence of stress among medical students (Hill et al., 2018). So, medical students are more likely to develop chronic pain disorders including FM and IBS. In this research, which is the first of its kind in Syria, the prevalence of IBS and FM will be evaluated among a sample of medical students at one Syrian medical school, and the results will be compared with a group of nonmedical students to find out if studying medicine associates with increasing prevalence of functional disorders.

## 2 MATERIALS AND METHODS

### 2.1 Study design, setting, duration, participants, and ethical considerations

We conducted an observational cross‐sectional study at Al‐Baath University, Homs, Syria, from 15 February to 15 March, 2023. The institutional research ethics board approval was obtained from Al‐Baath University before performing any study procedure (approval No.54658). The participants were undergraduate students, both medical and nonmedical, at Al‐Baath University, ranging in age from 18 to 30 years. The study included all individuals who consented to participate, each responder was given an informed consent form at the first page before filling out the survey. The participants’ identities are kept confidential.

### 2.2 Sample size calculation

Assuming an FM prevalence rate of 11.8% as reported in a previous study by Alzabibi et al. (2022), this study required 800 participants to detect a similar prevalence rate with 5% deviation and 95% confidence level. Sample size was calculated using OpenEpi online calculator according to calculation equation mentioned by Charan and Biswas (2013). Sampling method was non‐randomized, non‐intentional (convenience sampling).

### 2.3 Variables and data collection

A structured self‐estimated electronic questionnaire developed by Google Forms was distributed using social media platforms (Facebook, WhatsApp, and Telegram). The questionnaire of the study involved sociodemographic characteristics, including age, gender, and BMI. Furthermore, participants were asked about smoking, faculty, and curricular year. The ACR 2016 modified screening tool was applied to calculate the prevalence rate of FM, widespread pain index, and symptom severity scale (SSS) are the two parts of the tool (Wolfe et al., 2016). The number of body parts in which the patient reported experiencing pain throughout the previous week is used to calculate the widespread pain index. The SSS evaluates the severity of common FM symptoms such as, fatigue, waking up feeling unrefreshed, cognitive issues, headache, abdominal pain/cramps, and depression over the last 6 months. Moreover, we used Fibromyalgia Rapid Screening Tool (FiRST) criteria to assess the prevalence rate of FM, which included six questions about various FM‐related aspects, including widespread pain, fatigue, pain characteristics, non‐painful abnormal sensations, functional somatic symptoms, and sleep, and cognitive issues (Perrot et al., 2010). The ROME IV (2016) criteria were used to assess the prevalence of IBS and determine its type (Rome Foundation). This screening tool requires that patients have experienced a recurrent abdominal pain on average at least once per a week during the previous 3 months and that this pain is associated with two or more of the following: abdominal pain related to defecation, a change in frequency of stools, or a change in the shape or appearance of the stool.

### 2.4 Statistical analysis

Statistical analysis was done using SPSS v25 statistics software for Windows. In all cases, a *p* value <.05 was considered statistically significant. Continuous variables were presented as median and interquartile range; not normally distributed. Categorical variables were presented as percentages and frequencies. Using the Kolmogorov–Smirnov test, numerical factors’ normality was checked. The chi‐squared test was used to compare categorical variables, whereas the Mann–Whitney test was used for continuous variables. Logistic regression model was used to investigate some possible risk factors affecting the development of FM and IBS, and the results were given as 95% confidence interval with odds ratio. We used kappa test to assess the compatibility between ACR and FiRST criteria.

## 3 RESULTS

The final sample size was 800 individuals, which consisted of 499 females (62.4%) and 301 males (37.6%). Of the 800 participants, 347 (43.4%) were studying medicine and 453 (56.6%) were studying in other colleges. The median age of the participants was 22 years, and the median BMI was 21.5 (Table 1). The prevalence of IBS in the study population was 26.8%. Overall, IBS‐C (constipation) was the most common subtype, accounting for 40.2% of all IBS patients in Al‐Baath University, with IBS‐M (mixed) representing 37.4%, IBS‐D (diarrhea) 15%, and IBS‐U (undefined subtype) 7.5%. The prevalence of IBS was higher among medical students (14%) compared to other colleges (12.8%) (*p* = .002) with no relationship to the academic year (*p* = .067). Nonsmokers had higher IBS prevalence than the smokers (19.8% vs. 7%, *p* = .005). The prevalence of IBS was also significantly lower among those with FM according to (ACR) compared to those without FM (1.6% vs. 25.1%, *p* = .025). The difference in IBS prevalence between males and females was (9.3% vs. 17.5%, *p* = .283), but this did not reach the statistical significance (Table 2). The logistic regression showed that medical students (*p* = .002; OR: 1.64, 95%CI: 1.19–2.24) were at a higher risk of having IBS (Table 4). The prevalence of FM according to ACR in the study population was 3.6%. Females had higher FM prevalence than males (3.1% vs. 0.5%, *p* = .007). The prevalence of FM was also higher in other colleges compared to medicine (2.3% vs. 1.4%, *p* = .547), but this did not reach statistical significance. However, there was a statistically significant relationship between the medical academic year and FM prevalence (*p* = .008), where first‐year students had the highest prevalence rate, whereas sixth‐year students had the lowest one (Table 3). The logistic regression showed that females (*p* = .012; OR: 3.91, 95%CI: 1.35–11.36) were at a higher risk of having FM (Table 4). The prevalence of FM according to FiRST in the study population was 14.2%. Cohen's kappa was run to determine the agreement between the two screening tools on diagnosing FM (*k* = .206, *p* < .001); kappa coefficient revealed a statistically significant slight agreement.

**TABLE 1:** Characteristics of the study population.

	Total sample *N* = 800
Age*	22 (20–23) years
BMI*	21.5 (19.8–24.2) kg/m^2^
Gender: Male Female	301 (37.6%) 499 (62.4%)
Smoking: Yes No	272 (34%) 528 (66%)
College: Medicine: First year Second year Third year Fourth year Fifth year Sixth year Other colleges	347 (43.4%) 30 (3.8%) 79 (9.9%) 84 (10.5%) 68 (8.5%) 23 (2.9%) 63 (7.9%) 453 (56.6%)
Fibromyalgia (ACR)	29 (3.6%)
Fibromyalgia (FiRST)	114 (14.2%)
IBS	214 (26.8%)

Abbreviations: ACR, American College of Rheumatology; BMI, body mass index; FiRST, fibromyalgia rapid screening test; IBS, irritable bowel syndrome.

**TABLE 2:** Distribution of irritable bowel syndrome prevalence by respondent characteristics.

	With IBS	Without IBS	*p* Value
	*N* = 214 (26.8%)	*N* = 586 (73.2%)	
Gender Males Females	74 (9.3%) 140 (17.5%)	227 (28.4%) 359 (44.9%)	.283^b^
BMI	22.1 kg/m^2^	21.5 kg/m^2^	.037^a^
Smoking Yes No	56 (7%) 158 (19.8%)	216 (27%) 370 (46.3%)	.005^b^
College Medicine Others	112 (14%) 102 (12.8%)	235 (29.4%) 351 (43.9%)	.002^b^
Medical academic year First year Second year Third year Fourth year Fifth year Sixth year	8 (1%) 25 (3.1%) 26 (3.3%) 29 (3.6%) 11 (1.4%) 13 (1.6%)	22 (2.8%) 54 (6.8%) 58 (7.2%) 39 (4.9%) 12 (1.5%) 50 (6.3%)	.067^b^
Fibromyalgia (ACR) Yes No	13 (1.6%) 201 (25.1%)	16 (2%) 570 (71.3%)	.025^b^

Abbreviations: ACR, American College of Rheumatology; IBS, irritable bowel syndrome.

^a^ Mann–Whitney test.

^b^ Chi‐squared test.

**TABLE 3:** Distribution of fibromyalgia (FM) prevalence by respondent characteristics.

	With FM	Without FM	*p* Value
	*N* = 29 (3.6%)	*N* = 771 (96.4%)	
Gender Males Females	4 (0.5%) 25 (3.1%)	297 (37.1%) 474 (59.3%)	.007^b^
BMI	20.6 kg/m^2^	21.6 kg/m^2^	.103^a^
Smoking Yes No	14 (1.8%) 15 (1.9%)	258 (32.3%) 513 (64.1%)	.098^b^
College Medicine Others	11 (1.4%) 18 (2.3%)	336 (42%) 435 (54.4%)	.547^b^
Medical academic year First year Second year Third year Fourth year Fifth year Sixth year	4 (0.5%) 1 (0.1%) 2 (0.3%) 2 (0.3%) 2 (0.3%) 0 (0%)	26 (3.3%) 78 (9.8%) 82 (10.3%) 66 (8.3%) 21 (2.6%) 63 (7.9%)	.008^b^
IBS Yes No	13 (1.6%) 16 (2%)	201 (25.1%) 570 (71.3%)	.025^b^

Abbreviations: BMI, body mass index; IBS, irritable bowel syndrome.

^a^ Mann–Whitney test.

^b^ Chi‐squared test.

**TABLE 4:** The logistic regression of the possible risk factors.

Variable	Outcome	OR	95% CI	*p* Value
Sex	Fibromyalgia (ACR)	3.91	1.35–11.36	.012
Studying medicine	Irritable bowel syndrome	1.64	1.19–2.24	.002

Abbreviation: ACR, American College of Rheumatology.

## 4 DISCUSSION

In this study, we aimed to evaluate the prevalence of FM and IBS among Al‐Baath University students. To our knowledge, this is the first study of its kind in Syria concerned with the prevalence of these functional disorders in University students. In addition to impairing their psychological and social functioning, FM and IBS have a negative impact on their personal, professional, and everyday lives (Türkoğlu & Selvi, 2020). In our study, the prevalence of FM in Al‐Baath University students was found to be 3.6%. The prevalence of FM between medical students was 1.4%, which is roughly consistent with other studies in different countries, 2% in Turkish medical students (Eyigor et al., 2008) and 9.6% in Saudi medical students (Samman et al., 2021). Our study suggested that there is an association between gender and FM prevalence, where females had higher FM prevalence compared to males with an odds ratio of 3.91 and this result was indicated in previous studies, (Branco et al., 2010; Mäkelä & Heliövaara, 1991). We found no statistically significant association between studying medicine and FM prevalence. Eyigor et al. (2008) reported that the prevalence of generalized soft tissue rheumatism GSTR including FM in medical students was similar to previous reports in the general population. However, Patel et al. (2021) suggested that medical students must be considered a “high‐risk” group for FM. Moreover, we found a statistically significant association between medical academic year and FM prevalence, where first‐year students had the highest prevalence rate, whereas sixth‐year students had the lowest one, maybe due to students’ ability to identify and create stress‐reduction strategies as they advance in their academic year. Abdulghani et al. (2011) found that the prevalence of stress is higher during the initial 3 years of study. However, Samman et al. (2021) suggested that there is no relationship between the academic year and FM prevalence. The prevalence rate of FM according to FiRST was 14.2%, and the level of agreement between the two FM screening tools was slight but significant, the small sample size, small percentage of positive responses, and components of each tool are the main possible factors for this slight agreement. The prevalence of IBS in Al‐Baath University was 26.8% and between medical students was 14%. In Lebanon, University students have lower prevalence rate 20% (Costanian et al., 2015). However, medical students in other countries have higher prevalence rate, 31.8% in Jeddah (Ibrahim et al., 2013), 34.2% in Egypt (Abdulmajeed et al., 2011), and 28.3% in Pakistan (Naeem et al., 2012). These variations can be caused by genetic, environmental, or a combination of both sources (Kang, 2005). The most common type was constipation and that was consistent with Costanian et al. (2015) study. We found a statistically significant association between studying medicine and IBS prevalence with an odds ratio of 1.64, Chu et al. (2012) reported that medical students have a higher risk of any FBD (functional bowel disorder) than science and engineering students. Wani et al. (2020) suggested that there is an increased prevalence of IBS among medical students. We found no relationship between the academic year and IBS prevalence, Grundmann and Yoon (2010) also found an insignificant difference between preclinical and clerkship students in IBS prevalence. However, Ibrahim et al. (2013) reported that the prevalence of IBS was higher among older students and those from higher academic levels, maybe due to increased clinical load. Our study found that nonsmokers have higher prevalence of IBS than smokers and that was consistent with Mahmood et al. (2020) study. However, Arishi et al. (2021) reported that tobacco smoking is a risk factor for IBS, the exact mechanism which explain the relationship between smoking and bowel disorders is still unclear; it is maybe explained by the alteration of gut microflora composition caused by smoking, as Wang et al. (2012) reported. The prevalence of IBS was higher in females than in males, but this difference was not statistically significant maybe due to small sample size, despite the fact that gender has been linked to an increased risk of developing IBS in previous studies (Arishi et al., 2021; Chu et al., 2012; Ibrahim et al., 2013). The following limitations may have an impact on how reliable our research is. First, the questionnaire was voluntarily self‐administrated suggesting a possibility of information bias. Second, as a cross‐sectional research, the current investigation did not take time into account, so we are unable to draw conclusions about causality from these results. Finally, participants who met the IBS or FM diagnostic criteria were not further evaluated to rule out other potential diseases. However, the current study will surely improve the epidemiological statistics on the prevalence of these functional disorders, IBS and FM, in Syria.

## 5 CONCLUSION

We conclude that there is an increased prevalence of IBS among medical students. Although there is a common pathogenetic mechanism for FM and IBS, the prevalence of FM did not show any relation to studying medicine. Our study was a cross‐sectional study, so we recommend additional prospective studies to assess whether studying medicine could be a risk factor for these disorders or not. As medical school has a demanding educational system with high academic expectations that could have an impact on future medical practice, we recommend further screening of medical students for disorders like IBS and FM as well as stress reduction due to the possible influence of studying medicine on causing these disorders.

STROBE checklist of observational cross‐sectional studies.

## AUTHOR CONTRIBUTIONS

**Mohanad Daher**: Conceptualization; investigation; writing—original draft; methodology; formal analysis; supervision; writing—review and editing. **Sara Abbas**: Formal analysis; investigation; writing—original draft. **Zainab Asaad**: Investigation; writing—original draft; data curation. **Karam Khalil**: Data curation; investigation; writing—original draft. **Ghania Jadid**: Supervision.

## CONFLICT OF INTEREST STATEMENT

The authors certify that there are no conflicts of interest with any financial organization regarding the material discussed in the manuscript.

### FUNDING INFORMATION

This study was not funded by any sponsor.

### PEER REVIEW

The peer review history for this article is available at https://publons.com/publon/10.1002/brb3.3445.

## Data Availability

The data used to support the findings of this study are currently available upon request. The request will be considered by the corresponding author.

## References

[brb33445-bib-0009] Abdulghani, H. M. , AlKanhal, A. A. , Mahmoud, E. S. , Ponnamperuma, G. G. , & Alfaris, E. A. (2011). Stress and its effects on medical students: A cross‐sectional study at a college of medicine in Saudi Arabia. Journal of Health, Population, and Nutrition, 29(5), 516–522. 10.3329/jhpn.v29i5.8906 22106758 PMC3225114

[brb33445-bib-0010] Abdulmajeed, A. , Rabab, M. A. , Sliem, H. A. , & Hebatallah, N. E. (2011). Pattern of irritable bowel syndrome and its impact on quality of life in primary health care center attendees, Suez governorate, Egypt. The Pan African Medical Journal, 9, 5. 22145053 10.4314/pamj.v9i1.71177 PMC3215527

[brb33445-bib-0032] Alzabibi, M. A. , Shibani, M. , Alsuliman, T. , Ismail, H. , Alasaad, S. , Torbey, A. , Altorkmani, A. , Sawaf, B. , Ayoub, R. , Khalayli, N. , & Kudsi, M. (2022). Fibromyalgia: Epidemiology and risk factors, a population‐based case‐control study in Damascus, Syria. BMC Rheumatol, 6(1), 62. 10.1186/s41927-022-00294-8 36310163 PMC9618353

[brb33445-bib-0029] Arishi, A. M. , Elmakki, E. E. , Hakami, O. M. , Alganmy, O. M. , Maashi, S. M. , Al‐Khairat, H. K. , Sahal, Y. A. , Sharif, A. A. , & Alfaifi, M. H. (2021). Irritable bowel syndrome: Prevalence and risk factors in jazan region, saudi arabia. Cureus, 13(6), e15979. 34336470 10.7759/cureus.15979 PMC8316899

[brb33445-bib-0007] Branco, J. C. , Bannwarth, B. , Failde, I. , Abello Carbonell, J. , Blotman, F. , Spaeth, M. , Saraiva, F. , Nacci, F. , Thomas, E. , Caubère, J.‐P. , Lay, K. L. , Taieb, C. , & Cerinic, M. M. (2010). Prevalence of fibromyalgia: A survey in five European countries. Seminars in Arthritis and Rheumatism, 39(6), 448–453. 10.1016/j.semarthrit.2008.12.003 19250656

[brb33445-bib-0021] Cabo‐Meseguer, A. , Cerdá‐Olmedo, G. , & Trillo‐Mata, J. L. (2017). Fibromyalgia: Prevalence, epidemiologic profiles and economic costs. Medicina Clinica, 149(10), 441–448. 10.1016/j.medcli.2017.06.008 28734619

[brb33445-bib-0015] Charan, J. , & Biswas, T. (2013). How to calculate sample size for different study designs in medical research? Indian Journal of Psychological Medicine, 35(2), 121–126. 10.4103/0253-7176.116232 24049221 PMC3775042

[brb33445-bib-0012] Chu, L. , Zhou, H. , Lü, B. , Li, M. , & Chen, M. (2012). [An epidemiological study of functional bowel disorders in Zhejiang college students and its relationship with psychological factors]. Zhonghua Nei Ke Za Zhi [Chinese Journal of Internal Medicine], 51(6), 429–432. 22943750

[brb33445-bib-0018] Costanian, C. , Tamim, H. , & Assaad, S. (2015). Prevalence and factors associated with irritable bowel syndrome among university students in Lebanon: Findings from a cross‐sectional study. World Journal of Gastroenterology, 21(12), 3628–3635. 10.3748/wjg.v21.i12.3628 25834330 PMC4375587

[brb33445-bib-0024] Creed, F. (2020). A review of the incidence and risk factors for fibromyalgia and chronic widespread pain in population‐based studies. Pain, 161(6), 1169–1176. 10.1097/j.pain.0000000000001819 32040078

[brb33445-bib-0006] Eyigor, S. , Ozdedeli, S. , & Durmaz, B. (2008). The prevalence of generalized soft tissue rheumatic conditions in Turkish medical students. Journal of Clinical Rheumatology, 14(2), 65–68. 10.1097/RHU.0b013e31816b1920 18391672

[brb33445-bib-0008] Grundmann, O. , & Yoon, S. L. (2010). Irritable bowel syndrome: Epidemiology, diagnosis and treatment: An update for health‐care practitioners. Journal of Gastroenterology and Hepatology, 25(4), 691–699. 10.1111/j.1440-1746.2009.06120.x 20074154

[brb33445-bib-0023] Hill, M. R. , Goicochea, S. , & Merlo, L. J. (2018). In their own words: Stressors facing medical students in the millennial generation. Medical Education Online, 23(1), 1530558. 10.1080/10872981.2018.1530558 30286698 PMC6179084

[brb33445-bib-0016] Ibrahim, N. K. R. , Battarjee, W. F. , & Almehmadi, S. A. (2013). Prevalence and predictors of irritable bowel syndrome among medical students and interns in King Abdulaziz University, Jeddah. Libyan Journal of Medicine, 8(1), 21287. 10.3402/ljm.v8i0.21287 24054184 PMC3779356

[brb33445-bib-0033] Ioachim, G. , Warren, H. J. M. , Powers, J. M. , Staud, R. , Pukall, C. F. , & Stroman, P. W. (2022). Altered pain in the brainstem and spinal cord of fibromyalgia patients during the anticipation and experience of experimental pain. Frontiers in Neurology, 13, 862976. 10.3389/fneur.2022.862976 35599729 PMC9120571

[brb33445-bib-0019] Jones, G. T. , Atzeni, F. , Beasley, M. , Flüß, E. , Sarzi‐Puttini, P. , & Macfarlane, G. J. (2015). The prevalence of fibromyalgia in the general population: A comparison of the American College of Rheumatology 1990, 2010, and modified 2010 classification criteria. Arthritis & Rheumatology, 67(2), 568–575. 10.1002/art.38905 25323744

[brb33445-bib-0005] Kang, J. Y. (2005). Systematic review: The influence of geography and ethnicity in irritable bowel syndrome. Alimentary Pharmacology & Therapeutics, 21(6), 663–676. 10.1111/j.1365-2036.2005.02396.x 15771752

[brb33445-bib-0022] Lacy, B. E. , & Patel, N. K. (2017). Rome criteria and a diagnostic approach to irritable bowel syndrome. Journal of Clinical Medicine, 6(11), 99. 10.3390/jcm6110099 29072609 PMC5704116

[brb33445-bib-0011] Lumley, M. A. , Cohen, J. L. , Borszcz, G. S. , Cano, A. , Radcliffe, A. M. , Porter, L. S. , Schubiner, H. , & Keefe, F. J. (2011). Pain and emotion: A biopsychosocial review of recent research. Journal of Clinical Psychology, 67(9), 942–968. 10.1002/jclp.20816 21647882 PMC3152687

[brb33445-bib-0001] Mäkelä, M. , & Heliövaara, M. (1991). Prevalence of primary fibromyalgia in the finnish population. BMJ, 303(6796), 216–219. 10.1136/bmj.303.6796.216 1884057 PMC1670522

[brb33445-bib-0025] Mahmood, K. , Riaz, R. , Ul Haq, M. S. , Hamid, K. , & Jawed, H. (2020). Association of cigarette smoking with irritable bowel syndrome: A cross‐sectional study. Medical Journal of The Islamic Republic of Iran, 34, 72. 33306053 10.34171/mjiri.34.72 PMC7711034

[brb33445-bib-0013] Naeem, S. S. , Siddiqui, E. U. , Kazi, A. N. , Memon, A. A. , Khan, S. T. , & Ahmed, B. (2012). Prevalence and factors associated with irritable bowel syndrome among medical students of Karachi, Pakistan: A cross‐sectional study. BMC Research Notes, 5, 255. 10.1186/1756-0500-5-255 22624886 PMC3434121

[brb33445-bib-0030] Patel, A. , Al‐Saffar, A. , Sharma, M. , Masiak, A. , & Zdrojewski, Z. (2021). Prevalence of fibromyalgia in medical students and its association with lifestyle factors—A cross‐sectional study. Reumatologia, 59(3), 138–145. 10.5114/reum.2021.106908 34538940 PMC8436804

[brb33445-bib-0034] Perrot, S. , Bouhassira, D. , & Fermanian, J. , Cercle d'Etude de la Douleur en Rhumatologie (CEDR) . (2010). Development and validation of the Fibromyalgia Rapid Screening Tool (FiRST). Pain, 150(2), 250–256. 10.1016/j.pain.2010.03.034 20488620

[brb33445-bib-0017] Qin, H.‐Y. , Cheng, C.‐W. , Tang, X.‐D. , & Bian, Z.‐X. (2014). Impact of psychological stress on irritable bowel syndrome. World Journal of Gastroenterology, 20(39), 14126–14131. 10.3748/wjg.v20.i39.14126 25339801 PMC4202343

[brb33445-bib-0035] Rome Foundation . (2016). Rome IV criteria. Rome Foundation. https://theromefoundation.org/rome‐iv/rome‐iv‐criteria/

[brb33445-bib-0004] Rosal, M. C. , Ockene, I. S. , Ockene, J. K. , Barrett, S. V. , Ma, Y. , & Hebert, J. R. (1997). A longitudinal study of students’ depression at one medical school. Academic Medicine, 72(6), 542–546. 10.1097/00001888-199706000-00022 9200590

[brb33445-bib-0031] Samman, A. A. , Bokhari, R. A. , Idris, S. , Bantan, R. , Margushi, R. R. , Lary, S. , Sait, R. M. , & Bawazir, Y. M. (2021). The prevalence of fibromyalgia among medical students at King Abdulaziz university: A cross‐sectional study. Cureus, 13(1), e12670. 33489631 10.7759/cureus.12670 PMC7805490

[brb33445-bib-0026] Sarzi‐Puttini, P. , Giorgi, V. , Marotto, D. , & Atzeni, F. (2020). Fibromyalgia: An update on clinical characteristics, aetiopathogenesis and treatment. Nature Reviews Rheumatology, 16(11), 645–660. 10.1038/s41584-020-00506-w 33024295

[brb33445-bib-0003] Styles, W. M. (1993). Stress in undergraduate medical education: The mask of relaxed brilliance. British Journal of General Practice, 43(367), 46–47. PMC1372296 8466773

[brb33445-bib-0027] Türkoğlu, G. , & Selvi, Y. (2020). The relationship between chronotype, sleep disturbance, severity of fibromyalgia, and quality of life in patients with fibromyalgia. Chronobiology International, 37(1), 68–81. 10.1080/07420528.2019.1684314 31687843

[brb33445-bib-0002] Veale, D. , Kavanagh, G. , & Fielding, J. F. , Fitzgerald, O. (1991). Primary fibromyalgia and the irritable bowel syndrome: Different expressions of a common pathogenetic process. British Journal of Rheumatology, 30(3), 220–222. 10.1093/rheumatology/30.3.220 2049586

[brb33445-bib-0014] Wang, H. , Zhao, J.‐X. , Hu, N. , Ren, J. , Du, M. , & Zhu, M.‐J. (2012). Side‐stream smoking reduces intestinal inflammation and increases expression of tight junction proteins. World Journal of Gastroenterology, 18(18), 2180–2187. 10.3748/wjg.v18.i18.2180 22611310 PMC3351767

[brb33445-bib-0028] Wani, F. A. , Almaeen, A. H. , Bandy, A. H. , Thirunavukkarsu, A. , Al‐Sayer, T. A. , Flah, A. , Fayed, K. , & Albalawi, M. M. (2020). Prevalence and risk factors of ibs among medical and nonmedical students in the jouf university. Nigerian Journal of Clinical Practice, 23(4), 555–560. 10.4103/njcp.njcp_512_18 32246665

[brb33445-bib-0020] Wolfe, F. , Clauw, D. J. , Fitzcharles, M.‐A. , Goldenberg, D. L. , Häuser, W. , Katz, R. L. , Mease, P. J. , Russell, A. S. , Russell, I. J. , & Walitt, B. (2016). 2016 Revisions to the 2010/2011 fibromyalgia diagnostic criteria. Seminars in Arthritis and Rheumatism, 46(3), 319–329. 10.1016/j.semarthrit.2016.08.012 27916278

[brb33445-bib-0036] Wolfe, F. , Smythem, H. A. , Yunus, M. B. , Bennett, R. M. , Bombardier, C. , Goldenberg, D. L. , Tugwell, P. , Campbell, S. M. , Abeles, M. , Clark, P. , Fam, A. G. , Farber, S. J. , Fiechtner, J. J. , Franklin, C. M. , Gatter, R. A. , Hamaty, D. , Lessard, J. , Lichtbroun, A. S. , Masi, A. T. , & Sheon, R. P. (1990). The American college of rheumatology 1990 criteria for the classification of fibromyalgia. Report of the multicenter criteria committee. Arthritis and Rheumatism, 33(2), 160–172. 10.1002/art.1780330203 2306288

